# Cerebral Small Vessel Disease: Current and Emerging Therapeutic Strategies

**DOI:** 10.14336/AD.2024.1515

**Published:** 2026-02-03

**Authors:** Yuewen Sun, Xiaoqian Song, Chenyang Jin, Yilong Peng, Jing Zhou, Xueping Zheng

**Affiliations:** Department of Geriatric Medicine, The Affiliated Hospital of Qingdao University, Qingdao, Shandong, China

**Keywords:** Cerebral small vessel disease, treatment, pharmacological treatment, stroke, older people

## Abstract

Cerebral small vessel disease (CSVD) is a common disease in older people, characterized by damage to intracranial microvessels, leading to cognitive decline, increased risk of stroke, and dementia. This review reviews the current therapeutic approaches for CSVD and the latest research advances, encompassing traditional pharmacological therapies, emerging targeted interventions grounded in pathophysiology, exploratory immune-related treatments, and advances in genetic research. In addition, the role of lifestyle modifications in disease management is discussed. The review emphasizes the importance of a holistic, personalized treatment strategy to improve outcomes. More clinical trials are needed to validate these treatments and optimize individualized treatment options for CSVD patients.

## 1. Introduction

Cerebral Small Vessel Disease (CSVD) predominantly affects the elderly, involving a range of microvascular abnormalities like white matter hyperintensities (WMH), lacunar infarctions, cerebral microhemorrhages (CMB), and cerebral atrophy [1]. Based on the Neuroimaging Reporting Standard for Vascular Changes (STRIVE), CSVD is classified into six types: arteriosclerosis/age-related, amyloid-related, inherited non-amyloid CSVD, inflammation/immune-mediated, venous collagen disease, and other types [2, 3]. Clinically, CSVD may present with diverse symptoms or remain asymptomatic in its early stage. As the disease progresses, it can cause significant neurological impairments, including cognitive decline, stroke, and dementia, posing considerable diagnostic and therapeutic challenges. The public health impact is substantial due to its high disability rate and associated healthcare costs [4].

**Figure 1.**
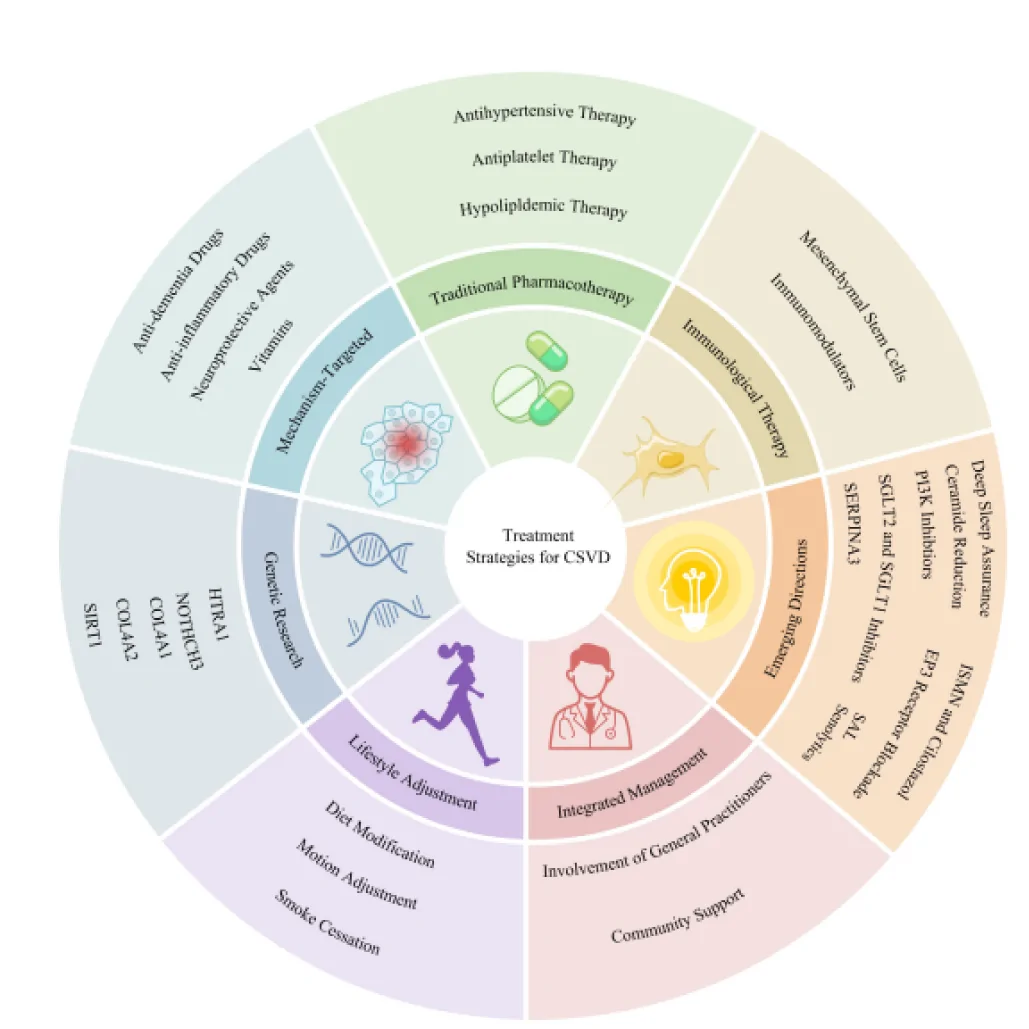
Current and emerging therapeutic strategies of CSVD.

The pathophysiological mechanism of CSVD is primarily driven by a complex interaction between endothelial dysfunction, blood-brain barrier (BBB) permeability, inflammation, and genetic factors [5]. These interactions gradually damage the brain's micro-vessels, leading to neurological dysfunction. Traditional pharmacological interventions, such as antihypertensive, hypolipidemic, and antiplatelet therapies, have been the mainstay of disease management. However, the multifactorial nature of the disease requires a broader, more integrated treatment approach. Due to the complexity of CSVD, no definitive treatment has been established, and many studies remain inconclusive. Effective strategies must target various underlying mechanisms. Understanding these advances is critical for clinicians and researchers aiming to improve patient outcomes and reduce the burden of this pervasive disease. This review aims to guide future research and clinical practice toward more effective, personalized treatment of CSVD (Fig. 1).

At present, no established criteria exist for assessing bleeding and stroke in patients with CSVD, highlighting the need for further research to enhance the scoring system. It can be further explored based on the study of large blood vessels. The risk score for atherosclerotic cardiovascular disease (ASCVD) over 10 years has proven valuable in predicting large vessel disease. A study found a positive correlation between the 10-year ASCVD score and CSVD as well as its MRI markers [6]. This indicates that ASCVD scores may serve as a useful instrument for assessing risk levels in individuals with CSVD.

## 2. Pharmacological characteristics and therapeutic strategies

Within the framework of pharmacotherapy for CSVD, its chronic and progressive nature demands sustained pharmacological approaches for long-term management. Given the substantial prevalence of CSVD, encompassing a quarter of all ischemic strokes, a 45% attribution to age-related dementia, and a WMH incidence surpassing 17% in individuals over 70 years old [7], any protracted treatment strategies must uphold economically viable costs at both the individual and societal levels [8, 9]. Effective therapeutic interventions for CSVD should predominantly rely on oral, transdermal, or nasal formulations or potentially long-acting injectable modalities. Taking into account the geriatric patient demographic, who are often prescribed a multitude of medications for various comorbidities such as cardiovascular disease prevention, arthritic conditions, and gastroesophageal reflux, treatment regimens with minimal drug-drug interactions and once- or twice-daily dosing schedules are favorable [10].

Considering the increasing worldwide demographic of the oldest segment of the population, the inclusion of patients aged 85 and above in future clinical trials is imperative to comprehensively evaluate the actual efficacy of treatment strategies in this elderly demographic. Until now, only a limited number of oldest-old patients have been enrolled in stroke prevention trials, which has constrained our profound understanding of their treatment outcomes. When setting clinical goals, the primary focus is on reducing the risk of both initial and recurrent strokes in individuals. Additionally, efforts are directed toward mitigating cognitive decline and physical impairments, including unstable gait, balance issues, and neuropsychological deficits. In terms of imaging assessments, the objectives include halting the progression of brain atrophy, microbleeds, and new lacunes, while also aiming to slow the advancement of white matter WMH [11].

To achieve these clinical and imaging objectives, precise lesion quantification techniques will be employed, carefully avoiding confounding factors in imaging measurements. Particular attention will be given to preventing the misincorporation of recurrent cortical or large subcortical infarcts into the assessment of WMH volume, ensuring that artificial increases in WMH burden are avoided [12]. Evaluating treatment efficacy should also encompass assessments of global or regional cortical and brainstem atrophy attributable to WMH and new lacunar ischemic strokes. These measures will provide a comprehensive understanding of the overall reduction in brain damage, contributing to improved patient outcomes.

## 3. Traditional pharmacotherapy

### 3.1 Antihypertensive Therapy

Among the various risk factors associated with cerebrovascular disorders, hypertension is a key and controllable factor, and the regulation of blood pressure within the normal range helps reduce vascular damage and further reduces the incidence of cerebrovascular diseases or CMB.

### 3.1.1 Evidence from Clinical and Epidemiological Studies

Clinical trials, such as the SPS3 and PRESERVE studies, have highlighted the potential benefits of intensive blood pressure management in reducing recurrent stroke risks [13, 14]. However, the impact on cognitive function and the identification of optimal blood pressure targets remains subjects of ongoing debate. Clinical trial data suggest that intensive antihypertensive therapy is effective in reducing the risk of repeat stroke and may reduce the occurrence of vascular cognitive impairment, and this approach seems to be safe in patients with advanced CSVD who may have impaired auto-regulation [15].Epidemiological studies further support that treatment of hypertension in midlife is more beneficial in preventing dementia than treatment in later life [16]. Hypertension's role in CSVD is further elucidated through imaging studies [17]. For example, higher systolic blood pressure (SBP) correlates with greater WMH volume and total brain atrophy. Patients maintaining SBP below 120 mmHg exhibit slower WMH progression compared to those with SBP below 140 mmHg, although brain volume reduction is slightly greater. These findings highlight the need for intensive patient-specific targets for blood pressure reduction [13].

### 3.1.2 Factors Influencing Antihypertensive Therapy Efficacy

Many factors influence the effect of antihypertensive therapy. Considerations including the extent and persistence of blood pressure lowering and the time point at which treatment is initiated may have an important impact on its effect. Additionally, the type of antihypertensive medication, its mechanism of action, and patient-specific factors can influence treatment efficacy. For instance, various antihypertensive agents may exhibit markedly different effects on blood-brain barrier (BBB) regulation. These differences may stem from the pharmacological characteristics of the drugs and the individual physiological state of each patient. In a study to determine whether classes of antihypertensive agents exert varying impacts on the function of microvasculature in individuals with small vessel disease, a meta-analysis revealed that various antihypertensive medications, including angiotensin-converting enzyme inhibitors (ACEIs), angiotensin receptor blockers (ARBs), calcium channel blockers (CCBs), and β-blockers (BBs), can effectively slow the progression of WMH. However, these drugs have a limited impact on brain atrophy, suggesting the involvement of pleiotropic mechanisms [18, 19]. In participants in a SPRINT-MIND (Systolic Blood Pressure Intervention Trial Memory and Cognition in Decreased Hypertension) trial, the use of ACEI and CCB reduced the progression of WMH regardless of blood pressure management and age, with ACEIs most consistent with WMH progression [20]. A Mendelian randomized study found that lowering blood pressure with CCB was significantly associated with lower WMH volume and significantly reduced the risk of BGPVS (basal ganglia perivascular space) and HIPPVS (hippocampal perivascular space), while other antihypertensive drugs did not affect CSVD imaging biomarkers. It is suggested that CCB is superior to other antihypertensive drugs in preventing or delaying the progression of CSVD [21]. This advantage may be attributed to CCB's unique mechanism of action. CCB can dilate cerebral vessels, increase cerebral blood flow, and reduce blood pressure variability. In contrast, BB or ACEI have minimal effects on cerebral blood flow, while CCB has shown better effects in reducing blood pressure variability, according to large-scale controlled trials [22, 23]. Nevertheless, large-scale studies regarding the prevention of CSVD with different types of antihypertensive agents are scarce. Whether the types of antihypertensive drugs have a differential effect on the clinical outcomes of patients with small vessel diseases needs further study. Antihypertensive treatment was beneficial to the progression of white matter hypersignal in small cerebral vascular disease but did not affect brain atrophy. Lower SBP levels are more effective for the progression of WMH [24]. There is insufficient evidence to prove the relationship between antihypertensive therapy and lacunar stroke and microbleeds.

In conclusion, antihypertensive therapy plays a pivotal role in CSVD management. Tailored approaches considering individual patient characteristics, drug-specific mechanisms, and optimal blood pressure targets are essential for maximizing therapeutic outcomes and improving patient quality of life.

### 3.2 Hypolipidemic Therapy

Statins, functioning as HMG-CoA reductase inhibitors, lower plasma concentrations of low-density lipoprotein cholesterol (LDL-C) by targeting the rate-limiting enzyme involved in cholesterol production, thereby significantly reducing the risk of atherosclerotic disease [25]. Given that elevated LDL-C represents a significant risk factor for CSVD and Alzheimer's disease (AD), the lipid-lowering effect of statins may theoretically be beneficial for CSVD [26].

A classic SPARCL (Stroke Prevention by Aggressive Reduction in Cholesterol levels trial) demonstrated that high-dose atorvastatin (80 mg/d) reduced the relative risk of stroke recurrence by 16% yet concurrently increased the risk of hemorrhagic stroke (intracerebral hemorrhage, ICH). This was particularly pronounced in patients with CSVD, with an excess risk of 4 to 5-fold [27]. This indicates that although statins are efficacious in reducing the risk of ischemic stroke, the risk of ICH must be meticulously evaluated when statins are employed in patients 3 with CSVD. Moreover, the association between statins and the risk of ICH is controversial in different studies [28].In recent times, proprotein convertase subtilisin/kexin type 9(PCSK9) inhibitors have emerged as a potent lipid-lowering class with potentially lower hemorrhagic risk. Statins have been shown to increase the risk of hemorrhagic stroke (HS) in a dose-dependent manner, depending on the type of cerebrovascular injury. In contrast, PCSK9 inhibitors do not appear to elevate the risk of HS. Therefore, PCSK9 inhibitors may be the new lipid-lowering drugs of choice for patients at high risk of HS, including those with a history of HS [29].

Furthermore, statins have shown certain effectiveness in managing WMH [30]. For example, low-dose rosuvastatin has been shown to slow the progression of WMH [31]. Nevertheless, the Pravastatin in the Elderly at Risk Study (PROSPER) did not demonstrate the efficacy of the drug in slowing the progression of WMH [32]. Despite the limited number of randomized controlled trials investigating the impact of lipid-lowering therapy on the progression of WMH and the inconsistency of their findings, the European Stroke Organization (ESO) guidelines recommend that statins should be considered in patients with CSVD, even if there are no other therapeutic indications for statins, especially in patients at higher risk of vascular events. However, the optimal dosage and the relative benefits and risks of standard versus intensive lipid-lowering strategies require further investigation. Moreover, while randomized clinical trials have not thoroughly evaluated the efficacy of statins in treating small vessel disease, most patients with CSVD exhibit an overall atherosclerotic cardiovascular risk [33]. This risk encompasses factors such as subclinical or diagnosed atherosclerosis, suggesting that statin therapy is a reasonable option for preventing atherosclerotic cardiovascular and cerebrovascular disease, irrespective of stroke considerations [34, 35]. There are no clear conclusions about the dosage use of statin therapy and the advantages and disadvantages of standard lipid-lowering and intensive lipid-lowering are still to be studied. In summary, the management criteria for patients with atherosclerosis can be referenced in the lipid-lowering therapy for CSVD. Currently, the use of statins following primary and secondary prevention guidelines for cardiovascular and cerebrovascular diseases is regarded as appropriate.

In conclusion, statin therapy holds a complex position in CSVD management. Moreover, the inconsistent evidence regarding statins' impact on WMH progression and the lack of clear dosage guidelines call for more large-scale, well-designed clinical trials. Future research should aim to clarify these uncertainties, enabling more precise and safer lipid-lowering strategies tailored to CSVD patients.

### 3.3 Antiplatelet Therapy

Large atherosclerotic (LAA) cerebral infarction is characterized by a large infarct caused by large atherosclerosis. This pathological change disrupts cerebral hemodynamics, reduces blood flow to distal small vessels, and causes small vessel injury, including white matter lesions and lacunar infarction associated with CSVD [36]. Conversely, small artery occlusion (SAO) type cerebral infarction is primarily caused by lesions in perforating or terminal arterioles, directly resulting in small lacunar infarcts and advancing small vessel disease [37]. Both LAA and SAO contribute to cerebrovascular damage at different vascular levels, and there may be interaction between these types during disease progression, which can jointly exacerbate CSVD [38]. Therefore, antiplatelet therapy is the basis of brain small vessel disease, and drugs such as aspirin, clopidogrel, and cilostazol are often recommended [39].

### 3.3.1 Risk-Benefit Assessment in Antiplatelet Therapy

Due to the dual risk of both ischemic events and hemorrhagic complications in CSVD, the benefit-risk ratio of antiplatelet therapy must be carefully evaluated [40]. Factors such as the patient's stroke history, small vessel disease etiology, and radiological findings are critical in tailoring treatment plans, as is balancing the risk of bleeding against potential therapeutic benefits [41]. Specific caution is necessary for patients with poor blood pressure control, high blood pressure variability, severe WMH, and multiple CMBs. For instance, in cases of cerebral hemorrhage linked to cerebral amyloid angiopathy (CAA), the recurrence rate is high, necessitating stringent blood pressure control, avoidance of emotional stress, and restriction of antiplatelet or anticoagulant therapies.

The timing of therapy resumption is influenced by factors like the severity and location of the hemorrhage. CMBs are pivotal in the treatment strategy of CSVD. Patients with co-existing CMBs and receiving single antiplatelet therapy (SAPT) showed a markedly higher risk of recurrent ischemic stroke. Conversely, in patients on dual antiplatelet therapy (DAPT), CMBs have been linked to a heightened risk of ICH [42]. Notably, the distribution of low-burden CMBs (<5 CMBS) had no significant effect on the recurrence of IS or ICH. For patients with acute IS and concurrent CMBs, DAPT may reduce the risk of IS recurrence but increases the likelihood of ICH. Subgroup analyses from randomized controlled trials (RCTs) focusing on lacunar stroke showed that SAPT lowered the relative risk of recurrent stroke by 22% compared to placebo (relative risk: 0.77, 95% confidence interval: 0.62-0.97). Currently, DAPT does not demonstrate significant clinical benefits over SAPT [43]. Given the low risk of secondary ischemic stroke in patients with CAA, antiplatelet drugs should be avoided in these patients [44]. For patients with hypertension, arteriolar lesions caused by hypertension are high-risk factors for ischemic stroke and cerebral hemorrhage. Atherosclerosis often co-occurs with hypertension and further increases the risk of ischemic stroke. Therefore, even if a patient has deep or subtentorial microbleeds, the benefit of oral antiplatelet therapy outweighs the risk of complications.

In patients with multiple CMB combined with atrial fibrillation, antiplatelet therapy requires more caution, and the benefits of anticoagulant therapy need to be further weighed against the risk of bleeding. Studies have suggested that such patients should consider reducing the antiplatelet dose or choosing an alternative to anticoagulant therapy. DOACs are typically safer than warfarin, with regular monitoring of bleeding risks and brain microbleeds advised [45, 46]. For patients with mild CMB burden (<5 CMBs), DOACs are generally safe and can be combined with low-dose antiplatelet therapy. In patients with moderate CMB burden (5-10 CMBs), DOACs may still be viable with careful bleeding risk assessment; delayed initiation (4-6 weeks) may reduce bleeding risk. For severe CMB burden (>10 CMBs or widespread distribution), intracranial hemorrhage risk is high, warranting extreme caution with anticoagulants, and non-pharmacological options like left atrial appendage closure may be considered [47, 48].

### 3.3.2 Timing for Initiating Antiplatelet Therapy

There are currently no standardized criteria for initiating antiplatelet therapy, with decisions typically made after the bleeding risk is mitigated. The timing of antiplatelet therapy for patients depends on their specific circumstances and risk factors. For patients with acute ischemic stroke, antiplatelet therapy should be started as early as possible, and aspirin as the preferred antiplatelet drug should be started within 48 hours of the onset of symptoms. Low-dose aspirin(75-300 mg) is the cornerstone of antiplatelet therapy, with the most abundant safety and efficacy data [49]. A study of post-ICH patients in China showed that aspirin use was not associated with an increased risk of recurrent ICH [50]. Therefore, aspirin is the primary treatment for secondary prevention of chronic ischemic stroke associated with CSVD.

In patients with mild acute ischemic stroke, DAPT may offer greater efficacy than monotherapy when initiated early (within 3 days), without substantially increasing the risk of intracerebral hemorrhage [51]. Clopidogrel and ticagrelor can be used as components of DAPT, but they should be started as early as possible and should not exceed 3 months. As a long-term secondary prevention strategy, they have been shown to increase the risk of cerebral hemorrhage, so long-term use is not recommended [52]. For patients at high risk of bleeding, such as those with prior cerebral hemorrhage and cerebral microbleeds, antiplatelet drugs should be started cautiously and after careful evaluation of the risks and benefits.

To sum up, many problems about the application of antiplatelet therapy still need to be studied. The CHA2DS2-VASc and HAS-BLED scores may be valuable for individualized treatment by assessing thrombosis and bleeding risks [53], while the S2TOP-BLEED score provides a comprehensive risk profile by factoring in patient health and comorbidities, aiding in antiplatelet regimen tailoring in clinical practice [54]. Among antiplatelet agents, cilostazol may offer a safer option for patients with coexisting WMH and multiple CMBs [55]. Additionally, research on newly diagnosed CSVD patients without dementia indicates that antiplatelet therapy is associated with reduced dementia risk, with clopidogrel potentially being more effective for dementia prevention than aspirin [56]. Nonetheless, further studies are needed to fully determine the comparative effectiveness of antiplatelet agents in dementia prevention.

## 4. Mechanism-Targeted Interventions

The blood-brain barrier (BBB) consists of a unique network of endothelial cells, exhibiting restricted transcellular and paracellular transport, encasing brain vessels to shield neurons from toxic substances. The transport function of endothelial cells is tightly controlled through interactions with astrocytes, pericytes, microglia, and the basement membrane, collectively forming neurovascular units that constitute the BBB. Astrocytes and pericytes wrap tightly around endothelial cells, supporting the link between blood flow and metabolic needs and releasing factors that strengthen and maintain BBB structure. CSVD plays a key role in cognitive decline and AD in aging populations [57].

**Table 1:** Research Progress of Novel Therapeutic Strategies.

Therapeutic Strategy	Main Mechanism	Preliminary Results	Research Stage
**AChEIs**	Inhibit acetylcholinesterase	Approved for Alzheimer's, further CSVD trials needed	Clinical use
**Memantine**	NMDA receptor antagonist	Moderate benefit in Alzheimer's, limited CSVD evidence	Clinical use
**Cilostazol**	PDE3 inhibitor	Mixed results in cognitive decline prevention	Phase III trials
**Isosorbide Nitrate (ISMN)**	NO donor	Ongoing, potential for cerebrovascular improvement	Clinical trials
**SGLT2/SGLT1 Inhibitors**	Regulate glucose, BBB protection	Ongoing, promising for CSVD management	Clinical trials
**Fingolimod**	Neuroinflammation modulation	Promising results for stroke/CSVD	Preclinical
**Edaravone Dexborneol (EDB)**	Anti-inflammatory, neuroprotective	Cognitive improvements in vascular dementia	Preclinical
**Epimedium Flavonoids (EF)**	Alleviate cognitive impairment	Reduces white matter lesions, cognitive improvements	Preclinical
**Salidroside (SAL)**	Endothelial regeneration	Improves BBB, reduces cognitive decline	Preclinical
**PI3K Inhibitors**	Improve neurovascular coupling	Promising for cognitive improvement	Preclinical
**Ceramide Reduction**	Inhibit ceramide production	Reduces neurovascular damage	Preclinical
**EP3 Receptor Blockade**	Modulate COX-2 pathway, CBF	Improves CBF, cognitive function in models	Preclinical
**Senolytics**	Eliminate senescent cells	Shows potential for restoring neurovascular function	Preclinical
**Deep Sleep Assurance**	Enhance SWA for BBB integrity	Diagnose and treat CSVD, BBB impairment	-
**SERPINA3**	Inflammatory biomarker for WMH	CSVD progression, therapeutic target	Preclinical
**Vitamins**	Regulate homocysteine, improve endothelial function	Treat hyperhomocysteinemia, reduce stroke risk	-

At present, although the pathogenesis of CSVD is still not clearly understood, previous studies have revealed that endothelial dysfunction and the enhancement of BBB permeability are linked to the disease's development, which is supported by experimental studies [58]. The most frequent initiating factor is endothelial dysfunction, which commonly disrupts BBB. This disruption represents a complex and intricate pathophysiological process that has been recognized as a key contributor to injury. In addition, many other factors may be involved, such as ischemia/hypoperfusion, obstruction of interstitial fluid return, inflammation, venous abnormalities, and genetic factors. These hypotheses provide an important framework for the study of CSVD therapy. Therefore, the current research direction for the treatment of CSVD is mostly based on this, such as antihypertensive drugs, anti-inflammatory drugs, anti-dementia drugs, vitamins, and some emerging therapeutic measures that can play a protective role. Effective treatment strategies require an integrated approach targeting a variety of underlying mechanisms. Currently, the clinical treatment of CSVD mainly relies on drugs to relieve symptoms, however, these drugs can relieve symptoms and have serious side effects. Therefore, the screening of drugs targeting cerebral microvascular endothelium, BBB, and neuroinflammation is the focus of clinical treatment. Table 1 integrates the main mechanisms and current research stages of various therapeutic strategies, covering both ongoing clinical trials and animal experimental studies, providing a reference for the development and application of novel treatments.

### 4.1 Anti-dementia Drugs

Recently, several studies have identified CSVD as a key contributor to the onset of senile dementia [59]. This condition often leads to vascular cognitive decline and dementia, possibly due to the concurrent presence and shared risk factors of vascular and neurodegenerative diseases [60]. Typically characterized by pathological changes in arterioles, capillaries, and venules, the resulting brain damage undermines cognitive function by disrupting the brain's intricate structural and functional networks [61].

Acetylcholinesterase inhibitors (AChEIs) effectively protect the neurotransmitter acetylcholine by inhibiting the activity of acetylcholinesterase, thereby enhancing signal transmission in cholinergic neurons. Currently, four AChEI drugs, including tacrine, rivastigmine, galantamine, and donepezil, have received approval for treating mild to moderate AD. Additionally, the non-competitive NMDA receptor antagonist memantine has shown effectiveness in the treatment of this condition [62].

Certain studies have suggested that AChEIs can stably improve cognitive function in patients with post-stroke cognitive decline and vascular dementia without significantly increasing the risk of adverse effects [63]. However, the effectiveness of AChEIs and memantine in preventing or treating CSVD remains uncertain, necessitating further investigation, especially regarding their potential limitations and long-term efficacy. Although memantine has demonstrated some clinical benefits in moderate to severe AD patients, regardless of whether they are also taking AChEIs, its effectiveness in mild dementia is not significant. These modest benefits are accompanied by side effects, including dizziness, nausea, vomiting, and diarrhea. If patients are willing to accept the risks, it is reasonable to trial the treatment, but if no clinically significant benefit is observed within 3 months, the drug should be discontinued. Moreover, the short-term nature of current clinical trials (mostly six months) limits a comprehensive assessment of their long-term efficacy and safety [64].

Regarding dementia-related behavioral and psychological symptoms, non-pharmacological management strategies, such as recognizing and addressing root causes and adopting personalized behavioral approaches, are often considered first-line treatment. The support for the effectiveness of psychotropic drugs in treating these symptoms is relatively limited, and they might be linked to higher rates of mortality, a greater risk of falls, and a decline in cognitive abilities. Therefore, future research needs to focus on optimizing non-pharmacological management strategies and exploring novel preventive and therapeutic approaches to improve the overall care of dementia patients [65].

### 4.2 Anti-inflammation Drugs

In studies in the field of CSVD, inflammation was identified as a significant risk factor [66]. The triggering of systemic inflammation can induce microglia to migrate to cerebrovascular regions, a behavior that may potentially undermine the BBB [67]. By delving into the interactions of clusters of CSVD-associated biomarkers, we were able to more accurately reveal the underlying pathological mechanisms. In addition, the central role of inflammation in multiple stroke causes is widely recognized, which provides strong support for the significant potential of anti-inflammatory therapy in secondary stroke prevention.

Several randomized controlled trials have demonstrated the positive effects of anti-inflammatory therapy in reducing the vascular risk of coronary artery disease and stroke risk. Two common classes of anti-inflammatory drugs, steroid and non-steroidal anti-inflammatory drugs (NSAIDs), have different properties in the treatment of CSVD. Considering that long-term use of steroid drugs may produce certain side effects, such as high blood pressure, high blood sugar, and osteoporosis. NSAIDs (e.g., aspirin, ibuprofen, naproxen) have become a more popular option due to their ability to selectively inhibit cyclooxygenase-2 (COX-2) activity and inhibit prostaglandin formation [68]. In addition, selective COX-2 inhibitors may increase the probability of vascular events in certain populations and should be used with caution [69].The effects and side effects of these anti-inflammatory drugs in patients with CSVD require further investigation.

In addition, a variety of drugs, including statins [32], prostacyclin (PGI2) analogues, nitric oxide (NO) donors, and phosphodiesterase (PDE) inhibitors have also been shown to exert anti-inflammatory effects by reducing cellular component activity and/or soluble biomarker levels, providing a variety of options for anti-inflammatory treatment of CSVD.

### 4.3 Neuroprotective Agents

Various neuroprotective anti-inflammatory agents, such as Fingolimod, natalizumab, and rituximab, have been employed in the treatment of neuroinflammatory conditions. This strategy is considered a promising treatment direction and is being investigated in depth through multiple randomized controlled trials. Edaravone dexborneol (EDB) stands out as a new neuroprotective agent capable of alleviating nerve damage from ischemic stroke. There is evidence that EDB, whose anti-inflammatory effects reduce neuronal loss and myelin damage, also significantly improves cognitive function [70].EDB enhances cognitive function, particularly learning and memory capabilities, in rodent models of vascular dementia. Additionally, it attenuates neuroinflammatory processes by mitigating the proliferation of glial cells, and effectively suppresses apoptosis and autophagy, potentially through modulation of the PI3K/Akt/mTOR signaling cascade [71]. Blocking the mutual circulation of free radicals and inflammation, and then playing a more effective neuroprotective effect, provides a new idea for the treatment of CSVD. In 914 patients with acute ischemic stroke within 48 hours, sublingual administration of EDB increased the proportion of patients with great functional outcomes at 90 days compared with placebo [72].

Epimedium flavonoids (EF), the primary bioactive constituents of Epimedium, a traditional Chinese medicinal herb, have shown potential as therapeutic agents for CSVD. The therapeutic potential of EF is attributed to its ability to alleviate cognitive impairment and white matter lesions caused by chronic cerebral hypoperfusion. Experiments suggest that this may be achieved by inhibiting the Lingo-1/Fyn/ROCK signaling pathway, as well as the activation of BDNF/TrkB, NRG-1/ErbB4 and downstream PI3K/Akt/CREB pathways in white matter [73].

### 4.4 Vitamins

In the study of multivitamins, B6, B12, and folic acid have attracted attention because of their significant regulatory effects on homocysteine (Hcy) metabolism [74]. Accumulating solid evidence shows that elevated serum Hcy levels are directly and indirectly linked to CSVD, in which endothelial dysfunction is a central factor [75]. Notably, hyperhomocysteinemia affects endothelial function at an early stage, through inflammatory pathways, oxidative stress, and epigenetic changes, and this effect is seen before small blood vessel damage and disease onset [76]. Therefore, hyperhomocysteinemia is seen as a key potential target for CSVD therapy. An in-depth study on secondary prevention of stroke has revealed the role of B vitamin supplements in reducing Hcy levels, significantly reducing the risk of stroke events and recurrence [77]. In addition, MRI-based neuroimaging sub-studies further support the association between B vitamins and reduced WMH changes in patients with severe WMH at baseline, providing strong evidence for the use of B vitamins in the treatment of CSVD.

## 4.5 Emerging therapeutic directions

### 4.5.1 Sodium-glucose cotransporter 2(SGLT2) and SGLT1 inhibitors

In addition to their role in glucose homeostasis, inhibitors of sodium-glucose cotransporters 2 (SGLT2) and 1 (SGLT1) may have potentially beneficial effects on circulating metabolic profiles that may contribute to attenuating the advancement of CSVD. Mendelian randomization (MR) studies have demonstrated that inhibiting SGLT2 and SGLT1 may confer protective effects against the development of CSVD. Notably, SGLT2 inhibition has been associated with a reduced risk of severe perivascular spaces, potentially through the modulation of 4-acylaminobutyrate levels and cholesterol metabolism. This inhibition also appears to enhance the integrity of the white matter microstructure, suggesting a multifaceted role of SGLT2 in mitigating CSVD progression [78].

### 4.5.2 Isosorbide nitrate (ISMN) and Cilostazol

Isosorbide nitrate (ISMN) is a nitric oxide (NO) donor that enhances the NO-cyclic guanosine phosphodiesterase PDE5 inhibitor pathway. Cilostazol is a phosphodiesterase 3′inhibitor that enhances the prostacyclin-cyclic AMP pathway and is widely used for stroke prevention in the Asia-Pacific region. Cilostazol has weak antiplatelet effects and may help prevent damage accumulation through myelin repair, endothelial stabilization, anti-inflammatory mechanisms, and neuroprotection. ISMN and Cilostazol demonstrate good safety and tolerability, with potential benefits in reducing recurrent stroke, dependency, and cognitive impairment post-lacunar stroke, along with other negative outcomes of CSVD. Thus, both agents warrant assessment in large phase 3 trials [79].In a randomized clinical trial, Cilostazol showed good tolerability but failed to prevent cognitive decline, indicating that further studies are needed to assess its efficacy [80].

### 4.5.3 Salidroside (SAL)

Extracted from Rhodiola rosea, Salidroside (SAL) has been extensively utilized for the treatment of coronary heart disease and myocardial ischemia over several decades. Research conducted earlier indicates that SAL is capable of diminishing arteriosclerosis, thereby countering ischemic brain injury [81].It has antioxidant, anti-inflammatory, anti-tumor and anti-radiation properties and can also be used as a neuroprotective agent [82, 83]. The findings from the study that mimicked CSVD in a rat model with progressive double-vessel occlusion (SHR-2vgo) revealed that the small molecule compound SAL, derived from botanical sources, can stimulate cerebral microvascular endothelial regeneration via the ItGb1-notch collaboration and mitigate the initial BBB damage associated with CSVD [84]. Consequently, SAL could be a promising therapeutic candidate for CSVD, offering a more cost-effective and less toxic treatment option.

### 4.5.4 PI3K Inhibitors

Longitudinal studies have shown that chronic PI3K inhibition also improved memory function in older Col4A1 mutant mice, and one experimental data suggests that PI3K inhibition is a promising therapeutic strategy for the treatment of deficient neurovascular coupling (NVC) and cognitive impairment [85].

### 4.5.5 EP3 Receptor Blockade

Given that long-term use of COX-2 inhibitors increases the risk of vascular disease and adverse side effects by suppressing endo prostacyclin production, modulating prostaglandins and their receptors downstream of the COX-2 pathway is proposed as a potential therapeutic approach. In COX-2-mediated inflammatory processes, prostaglandin E2 (PGE2) exerts a pivotal role through its interaction with e-prostaglandin (EP) receptors (EP1-4) [86]. Notably, EP3 gene deletion significantly mitigated ECM overexpression in cerebral arterioles in a CSVD rat model, leading to improvements in cerebral blood flow (CBF) and cognitive function. This effect may be closely associated with the downregulation of the TGF-β1/Smad signaling pathway [87]. Therefore, EP3 receptor blockade emerges as a promising therapeutic strategy for CSVD. While selective EP3 receptor antagonists, including L798106, ONO-AE3-240, and DG-041, exhibit potential therapeutic benefits, their further application necessitates rigorous preclinical and clinical investigations [88].

### 4.5.6 Senolytics

As the body ages, senescent cells, including endothelial cells, astrocytes and microglia, accumulate in the neurovascular unit, which leads to the damage of neurovascular function, the imbalance of cerebral blood flow and the damage of the blood-brain barrier [89]. Spurred by these findings, senolytics, a class of therapeutic drugs that excel at selectively eliminating senescent cells, has generated interest for their ability to restore tissue function and mitigate age-related lesions in preclinical Settings [90-92]. An experimental study in elderly mice showed that senolytics could work by counteracting the harmful effects of aging on the brain's microvessels, suggesting that anti-aging interventions may be a viable therapeutic route to prevent or mitigate the consequences of aging-related CMBs and thus to treat CSVD [93].

### 4.5.7 Ceramide reduction

The increase in ceramide has a wide range of effects on various brain cells, including endothelial cells, neurons, and microglia. Thus, strategies to reduce ceramide synthesis, such as regulating sphingomyelin activity or targeting key enzymes in its metabolism, may be beneficial [94].The serine palmitoyl transferase (De novosynthesis pathway) may provide a new and promising therapeutic pathway for the prevention or treatment of cerebrovascular injury-related diseases. Smpd3, a gene encoding neutral sphingomyelins, has been identified as a novel target gene regulated by HIF-1αand plays a critical role in ceramide production. Lentivirus-mediated SMPD3 injection into epididymal adipose tissue reversed ceramide reduction in adipocytes and negated the improvement of atherosclerosis in mice lacking HIF-1αin adipocytes [95]. Therefore, inhibiting HIF-1α may offer a novel approach to slowing down the progression of atherosclerosis.

### 4.5.8 Deep Sleep Assurance

New insights into sleep and cognition suggest that sleep is crucial in maintaining central nervous system health and fighting CSVD, and lack of sleep may exacerbate the accumulation of metabolites and harmful substances in the brain, damaging BBB [96, 97]. Recent studies suggest that slow wave activity (SWA, 0.5-4 Hz) occurring during deep sleep phases may reflect the lymphatic pathology, BBB permeability, and memory impairments of AD, providing a new perspective for the diagnosis and therapeutic management of AD [98]. In addition, SWA's non-invasive and bedside detection potential makes it a rapid diagnostic tool for assessing BBB permeability and offers promising treatment strategies for amyloid-related CSVD and BBB impairment [99].

### 4.5.9 Serine protease inhibitor A3 (SERPINA3)

The exact link between different inflammatory biomarkers and the progression of CSVD is not fully understood. Nevertheless, a study involving 79 patients with acute ischemic stroke revealed that elevated levels of serine protease inhibitor A3 (SERPINA3) were significantly associated with the severity of WMH [100]. This association was substantiated through multiple regression analysis and restricted cubic spline regression, indicating that SERPINA3 may serve as a potential biomarker for CSVD. This finding further underscores the roles of inflammation and endothelial dysfunction in the pathogenesis of CSVD. Future research will aim to elucidate the mechanisms by which SERPINA3 influences CSVD and to develop novel therapeutic strategies targeting this biomarker.

## 5. Immunological Therapy

Mesenchymal stem cells (MSCs) are stromal cells capable of multi-lineage differentiation and self-renewal. In recent research, mesenchymal stem cell therapy has emerged as an emerging strategy in the treatment of central nervous system diseases due to its unique paracrine properties and immunomodulatory capabilities [101]. Gene expression analysis showed that transfused mesenchymal stem cells activated transforming growth factor-β and angiopoietin 1 signaling pathways and promoted remodeling of the microvascular system [102]. Studies have shown that Aβ accumulation, AQP4 polarity disturbances, and neuroinflammation may be linked to CSVD. The modulatory role of MSCs in these processes implies a therapeutic potential for CSVD [103]. The therapeutic advantages of MSC stem from its multifunctional differentiation potential and ability to secrete A variety of bioactive factors to induce damaged tissue repair, not only targeting the classic pathological features of AD, but also acting on astrocytes, improving Aβ degradation, reducing neuroinflammation, and alleviating synaptic dysfunction [104, 105]. MSCs can alleviate neuroinflammation by promoting AQP4 polarity and STAT6 pathways. However, the exact mechanism of action of MSC in CSVD has not been clarified, so further animal studies are needed to determine the exact underlying mechanism of MSC therapy in CSVD. In addition, the therapeutic efficacy of MSCs and the types of MSCs suitable for CSVD research need to be confirmed by further clinical studies.

Considering that in white matter lesions caused by CSVD, glial cells react to pathological processes in various manners. In particular, notable alterations include the loss of myelin and oligodendrocytes, as well as glial activation of astrocytes, microglia, and NG2 glia [106]. The protective effects of various immunomodulatory molecules secreted by these cells, including chemokines, cytokines, and complement and their receptors, provide a new feasibility for the immunotherapy of diseases [107]. Granulocyte colony-stimulating factor (G-CSF), expressed by neurons, immune cells, and endothelial cells, demonstrates neuroprotective effects. It promotes neuronal regeneration, inhibits neuronal apoptosis, mobilizes hematopoietic stem cells (HSCs), modulates inflammatory cytokines, and stimulates angiogenesis [108]. An experiment has shown that exogenous G-CSF can promote endothelial cell repair and reduce white matter damage in spontaneously hypertensive rats, thus improving non-spatial cognitive ability in patients with cardiovascular disease [109]. Given the similarities in inflammatory mechanisms and clinical manifestations between multiple sclerosis (MS) and CSVD, immunomodulatory strategies used in MS may serve as a reference for CSVD treatment and prevention [107]. Compared with MS treatment strategies, the results from proof-of-concept clinical trials assessing Fingolimod for acute ischemic stroke and cerebral hemorrhage, as well as the evidence affirming the efficacy of Natalizumab in acute ischemic stroke [110], support the potential feasibility of immunological intervention in the treatment of CSVD, thus further verifying the scientific nature of this idea. Immunotherapy aims to mitigate inflammation and improve clinical outcomes in patients.

## 6. Genetic Research

In the evolving field of CSVD genetics research, NOTCH3, HTRA1 (high-temperature requirement A serine peptidase 1), COL4A1 (collagen type IV alpha 1), and COL4A2 mutations have emerged as key genetic drivers of single-gene adult-onset CSVD [111-114]. Among these, NOTCH3 mutations are predominantly associated with CADASIL, the most common monogenic form of CSVD. These mutations typically alter cysteine residues in the epidermal growth factor-like repeat domains of the NOTCH3 protein, leading to pathological vascular changes.

Current gene therapy approaches for CADASIL focus on mitigating the downstream effects of NOTCH3 mutations, particularly the loss of vascular smooth muscle cells (VSMCs) and pericytes, which play central roles in disease pathogenesis [115]. These strategies include growth factor administration, antisense oligonucleotide-mediated NOTCH3 cysteine-specific exon skipping, and agonist NOTCH3 antibodies [116-118]. These methods aim to pave the way for future targeted interventions in CADASIL. Additionally, knocking down the NOTCH3 gene in VSMCs with short hairpin RNA (shRNA)has led to actin cytoskeleton changes resembling those observed in CADASIL VSMCs, providing valuable insights into the potential of shRNA-mediated silencing of the mutated NOTCH3 gene to mitigate its deleterious gain-of-function effects [105].Active immunotherapy targeting NOTCH3 extracellular domain (NOTCH3ECD) aggregation has shown favorable tolerability, reducing NOTCH3 deposition in brain capillaries, enhancing microglia activation, and decreasing serum NOTCH3ECD levels in the mouse model carrying the human NOTCH3 R182C mutation [119]. This suggests that targeted vaccines against gene expression products are a therapeutic strategy with potential clinical application. Also, the researchers have used omics techniques to identify several promising drug targets, such as TIMP3 (belonging to the TIMP family, which are metalloproteinase inhibitors) and vitreous connection, which are involved in NOTCH3 deposition [120].

Currently, research on NOTCH3 in CSVD is a leading focus, with other related genetic studies also advancing. For example,through rigorous Mendelian randomization, a group of druggable genes, including ALDH2, KLHL24, ADRB1, BTN3A2, and EFEMP1, have recently been identified that are causally linked to the pathogenesis of cerebrovascular disease, providing new targets for therapeutic intervention, and highlighting the potential for personalized medicine in the treatment of CSVD [121].Experiments in mice demonstrated that COL4A1/COL4A2 variants contribute to early-onset CSVD and ICH through endothelium dependent hyperpolarization (EDH) dysregulation and decreased type IV collagen levels, highlighting EDH and type IV collagen as potential therapeutic targets for CSVD [122].Also, CSVD is related to the pathological mechanism of AD [123]. A study revealed differences in gene expression between AD and progressive and non-progressive CSVD by analyzing the gene expression profiles of GSE63060 (AD) and GSE162790 (CSVD). A total of 146 common differentially expressed genes (DEGs) were identified, primarily enriched in endocytosis and oxytocin signaling pathways. The results showed that SIRT1 plays an important role in common DEGs, and the SIRT1 gene may represent a promising therapeutic target for the treatment of AD as well as CSVD [124].This approach facilitates additional studies on the connection between CSVD and genes.

Together, these strategies underscore a multifaceted approach to CSVD gene therapy, integrating exon-skipping techniques, gene silence, immunotherapy, and molecular targeting. While these interventions show promise in preclinical settings, further research and clinical trials are required to validate their efficacy and safety for translational application in CSVD management. While these inherited CSVD syndromes can be confirmed with genetic testing, they are uncommon, and the cost of testing is an additional expense for patients.

## 7. Lifestyle Modification

Studies have indicated a possible causal relationship between cardiovascular risk factors and the etiology of CSVD [125]. In particular, high blood pressure (especially diastolic blood pressure) and high body mass index (BMI) are strongly associated with CSVD [126].In the management of patients, in addition to drug treatment, lifestyle adjustment plays a more important role. The measure covers everything from diet to exercise habits to quitting smoking. It is the most economical and efficient treatment to control the risk factors of the disease through the change of lifestyle and prevent the disease in the non-disease stage.

### 7.1 Diet Modification

Recent research continues to underscore the value of a diet low in salt, fat, and high in fiber.Evidence indicates that a low-sodium diet effectively reduces blood pressure, thereby mitigating the detrimental effects of hypertension on cerebral vasculature [127]. Moreover, reducing the intake of saturated and trans fats while incorporating something rich in omega-3 fatty acids, such as deep-sea fish and walnuts, can lower blood lipid levels and improve vascular health. The role of antioxidant-rich foods has also gained more traction. Additionally, the consumption of foods high in antioxidants, like blueberries and spinach, can counteract the damage caused by free radicals to brain vessels [128].

Certain superfoods may enhance neuronal function and synaptic plasticity by activating key biological pathways critical for brain health and mental well-being. For instance, diets rich in choline, magnesium, omega-3 fatty acids, specific amino acids, vitamin D, vitamin B, and plant or seaweed derivatives not only enhance brain health and maintain mental acuity but may also reduce the risk of neurodegenerative and psychiatric disorders [129]. In the case of spirulina microalgae, its blood pressure-regulating properties have been linked to stroke prevention, though further clinical studies are required to elucidate its neuroprotective mechanisms despite promising preclinical and initial clinical findings [130].

### 7.2 Motion Adjustment

The benefits of moderate aerobic exercise for cardiovascular health have been further corroborated by recent investigations. Moderate aerobic exercise, like brisk walking, swimming, and cycling, offers numerous benefits for cardiovascular health. It enhances cardiovascular function, increases blood flow to the brain, and significantly reduces the risk of small vessel diseases. Interestingly, even a small amount of low-intensity physical activity (LPA)—less than 90 minutes a week—can lower the risk of an initial ischemic stroke [131]. This makes LPA not only more practical but also more sustainable and easier to promote widely. However, patients must choose the right type and intensity of exercise suited to their health conditions to avoid any potential harm from overexertion.

### 7.3 Smoke Cessation

Recent long-term follow-up studies have deepened our understanding of smoking as a formidable, independent risk factor for CSVD. There is already evidence that smoking is associated with new cases of CSVD. It impacts vasoconstriction, triggers hypertension, and speeds up atherosclerotic processes. A 10-year follow-up study on smoking and quitting age shows that smokers are at a higher risk of stroke compared to those who have never smoked [132, 133]. This risk rises as the smoking duration lengthens. Quitting smoking can lower the risk of stroke. In particular, quitting at a younger age is especially beneficial. Given these findings, CSVD patients must be vehemently counseled to quit smoking and shun passive smoke exposure, as any form of nicotine intake can perpetuate vascular damage.

## 8. Integrated management

Family and community support are essential in preventing and managing CSVD. Tools like the Mini-Mental State Examination (MMSE) [134], and Montreal Cognitive Assessment (MoCA) [135] are effective for screening dementia and mild cognitive impairment in older people. Leveraging these tools within community settings facilitates comprehensive cognitive evaluations and enhances early detection.

Advancements in smart medical technologies have paved the way for personalized treatments tailored to individual needs. General practitioners, collaborating closely with families and communities, play a pivotal role in this effort [136]. This includes organizing health seminars, providing free medical consultations, and working with community health facilities to provide comprehensive medical services to patients. This integrated approach provides patient-centered, ongoing services that help reduce the risk of recurrence of CSVD. More important is the need to raise public awareness and self-management skills.

Recent advancements in deep learning have significantly improved the reliability and efficiency of automatic CSVD imaging quantification. Tools like AccuBrain® exhibit high accuracy, reproducibility, and efficiency, enabling automated measurements of brain atrophy [137, 138] and WMH [139]. These tools hold promises for broader quantification of CSVD features. These technological innovations support deeper investigations into CSVD mechanisms, facilitate precision medicine, and contribute to enhanced patient outcomes.

Despite these advancements, significant gaps remain between existing quantification techniques and their clinical adoption, primarily due to integration challenges and limited accessibility for clinicians. Addressing these challenges necessitates the development of validated, user-friendly tools capable of robustly quantifying multiple CSVD features, bridging the gap between research and practice.

Personalized medicine plays a critical role in CSVD management, emphasizing tailored treatment strategies. For instance, genetic markers predicting treatment responses can guide therapy selection, with individuals carrying endothelial function-related genetic variants benefiting more from vasoprotective interventions. Furthermore, lifestyle factors, such as diet and physical activity, are integral to treatment planning. For managing comorbidities such as diabetes, a comprehensive approach is necessary to mitigate potential drug interactions and ensure thorough care. The coexistence of multiple diseases corresponds to diverse foci, underscoring the importance of individualized treatment. This necessitates a more extensive assessment and investigation.

## 9. Research dilemmas

There are many difficulties that need to be overcome in the further exploration of current research on treatment strategies, and researchers are often in a dilemma. To enhance the validity of claims regarding efficacy and safety for treatment strategies that are controversial or not broadly acknowledged, it is imperative to integrate more systematic reviews and meta-analyses.

Economic and ethical considerations are pivotal in the deployment of emerging CSVD therapies. The cost-effectiveness of these treatments must be evaluated, considering the high costs associated with research, development, and production. For instance, gene therapies require substantial investment in sequencing, vector development, and specialized infrastructure. The assessment of cost-effectiveness should weigh the potential benefits, such as reduced stroke recurrence and prevention of dementia, against the financial burden. If the clinical benefits are substantial and long-lasting, a higher upfront cost may be justified.

Ethical concerns related to gene therapies include the potential for off-target effects and unforeseen consequences on the human genome. The long-term implications for future generations and the possibility of alterations in germline cells are also of concern. Furthermore, the equitable distribution of these costly therapies is an ethical issue, as financial constraints may limit access, leading to disparities in healthcare provision. Addressing these economic and ethical challenges is critical for the sustainable and equitable advancement of emerging CSVD treatments.

## 10. Conclusion

This review summarizes recent advances in CSVD treatment and directions for future sustained research. As the population ages, the prevalence of CSVD among older adults is rising, making research on its prevention and treatment highly valuable. The existing primary strategies for CSVD prevention and treatment are symptomatic drug treatment and controlling the disease's risk factors, especially for patients with clinical manifestations. This paper clarifies the efficacy and limitations of current pharmacological interventions. Meanwhile, it deeply explores the advantages and disadvantages of emerging therapeutic approaches, highlighting the significance of lifestyle modifications and individualized management. Despite the progress achieved, discussions regarding therapeutic limitations and potential adverse effects are insufficient, so more clinical trials are required. Future research should initiate with a multidisciplinary exploration of pathogenesis. This will enable early diagnosis, guide clinical decision-making, boost preventive measures, and ease the burden on patients and their caregivers. Incorporating a geriatric perspective allows for a more effective response to the challenges CSVD brings to the public, especially in an aging society.
